# Deep Reinforcement Learning-Based One-to-Multiple Cooperative Computing in Large-Scale Event-Driven Wireless Sensor Networks

**DOI:** 10.3390/s23063237

**Published:** 2023-03-18

**Authors:** Zhihui Guo, Hongbin Chen, Shichao Li

**Affiliations:** Key Laboratory of Cognitive Radio and Information Processing, Ministry of Education, Guilin University of Electronic Technology, Guilin 541004, China

**Keywords:** wireless sensor networks, cooperative computing, dynamic clustering, deep reinforcement learning

## Abstract

Emergency event monitoring is a hot topic in wireless sensor networks (WSNs). Benefiting from the progress of Micro-Electro-Mechanical System (MEMS) technology, it is possible to process emergency events locally by using the computing capacities of redundant nodes in large-scale WSNs. However, it is challenging to design a resource scheduling and computation offloading strategy for a large number of nodes in an event-driven dynamic environment. In this paper, focusing on cooperative computing with a large number of nodes, we propose a set of solutions, including dynamic clustering, inter-cluster task assignment and intra-cluster one-to-multiple cooperative computing. Firstly, an equal-size *K*-means clustering algorithm is proposed, which activates the nodes around event location and then divides active nodes into several clusters. Then, through inter-cluster task assignment, every computation task of events is alternately assigned to the cluster heads. Next, in order to make each cluster efficiently complete the computation tasks within the deadline, a Deep Deterministic Policy Gradient (DDPG)-based intra-cluster one-to-multiple cooperative computing algorithm is proposed to obtain a computation offloading strategy. Simulation studies show that the performance of the proposed algorithm is close to that of the exhaustive algorithm and better than other classical algorithms and the Deep Q Network (DQN) algorithm.

## 1. Introduction

Emergency event monitoring is widely used in Wireless Sensor Networks (WSNs), such as fault detection, target tracking, disaster alarms and other engineering applications. In classical WSNs, the monitored data are sent back to the cloud center for processing, but the transmission of massive data will cause excessive delay and energy consumption. Recently, sensors have gained greater computing capacity through technical advances in Micro-Electro-Mechanical Systems (MEMS), which makes local processing of the monitored data possible by exploiting a sensor’s own computing resources [1].

However, it is inadequate for a single sensor node to process urgent computation tasks independently due to its limited computing capacity and battery energy. Fortunately, the advances in edge computing and cooperative computing have opened new avenues to address this challenge. Edge computing is a new paradigm that moves computation tasks and services from the cloud center down to the edge servers [2]. Different from edge computing, in which computation tasks are offloaded to edge servers, cooperative computing pays more attention to mining the computing resources of peer terminals to complete computation tasks. Through the cooperative computation between sensor nodes, some lightweight computation tasks can be handled locally [3]. Limited by a battery energy of nodes, if a node undertakes collaborative tasks successively, the node will die (run out of energy) quickly, and this will result in coverage holes in WSNs [4]. In our previous study, a one-to-one cooperative computing scheme based on sleep scheduling was proposed, which can complete more of the lightweight tasks and prolong the network lifetime by activating different sensors alternately in WSNs [5]. However, in one-to-one cooperation, the task node (the node that receives the computation tasks) can only divide the computation tasks into two parts, which are completed by the task node and the cooperative node, respectively. Limited by the computing capacity of a single cooperative node, it is difficult for one-to-one cooperation to complete complex and urgent tasks. In large-scale WSNs, there are a large number of redundant nodes due to dense deployment. Leveraging redundant nodes to participate in cooperative computing can not only alleviate the coverage hole problem caused by the death of some cooperative nodes but also make full use of local computing resources. Therefore, this paper proposes a one-to-multiple cooperative computing scheme, which utilizes the computing capacities of redundant nodes to complete more complex computing tasks locally. In one-to-multiple cooperation, the task node can divide the computation tasks into several parts and complete the computation tasks through the distributed parallel computing of multiple cooperative nodes.

Nevertheless, one-to-multiple cooperative computing for large-scale event-driven WSNs still faces some challenges. First, if too many redundant nodes participate in cooperation at the same time, it is not only easy to cause network congestion but also may waste a lot of energy and computing resources. Second, the randomness of event location and computation tasks makes it difficult to determine the optimal computation offloading scheme. Third, a large number of nodes participate in cooperative computing, which brings new challenges to the energy consumption balance.

To tackle the above challenge, we propose a set of solutions, including dynamic clustering, inter-cluster task assignment and intra-cluster multi-node cooperative computing. First, we propose an equal-size *K*-means clustering algorithm, which activates nodes around the event location and then divides active nodes into several clusters. Then, through inter-cluster task assignment, every computation task of events is alternately assigned to the cluster heads. Next, every cluster head, which can be regarded as a task node, needs to complete its own tasks by intra-cluster one-to-multiple cooperative computing.

For the intra-cluster one-to-multiple cooperative computing, in order to make each cluster efficiently complete the computation tasks within the deadline, we need to design a computation offloading strategy for each cluster. It is a complicated decision problem that is difficult to handle using traditional optimization algorithms. This is because the randomness of emergency events and dynamic clustering determines that the event-driven WSN is a highly indeterminate system, and the number of cooperative nodes and the ratio of computation offloading to each cooperative node are also variable.

Fortunately, the development of artificial intelligence technology provides new ideas for solving such problems. Q learning, a model-free reinforcement learning algorithm, has been successfully used to solve this kind of optimization problem in an indeterminate environment [6]. However, with the increase in the state space and action space, it becomes difficult in Q learning to express the Q-value in a table [7]. In solving high-dimensional state and action space decision problems, Deep Reinforcement Learning (DRL) algorithms, such as DQN, have been proven to be more effective than traditional reinforcement learning. However, the classical DQN algorithm [8] can only deal with discrete action space. For continuous action space, the discrete action space defined manually in DQN can no longer be fine enough. Therefore, we propose a DDPG-based one-to-multiple cooperative computing algorithm. DDPG is a kind of DRL algorithm in which the action space is continuous. The DDPG algorithm adopts the actor–critic schema. The actor network is used to represent the offloading action policy, and its output is continuous values, which means that the task can be offloaded at any ratio. The critic network is used to evaluate the quality of actions by estimating the *Q* value. Simulation studies show that the performance of the proposed algorithm is close to that of the exhaustive algorithm and better than the classical algorithms and the DQN algorithm. The contribution of this paper mainly includes the following aspects:

- A large-scale event-driven WSN model with one-to-multiple cooperative computing is constructed. Different from the cloud-assisted approach, this model focuses on using the computing capacities of sensor nodes to handle event monitoring locally.
- An equal-size *K*-means clustering algorithm is proposed. As a result of clustering, the number of nodes in each cluster is almost the same, rather than arbitrary, which is helpful for the execution and expansion of the subsequent offloading algorithm.
- A DDPG-based one-to-multiple cooperative computing algorithm is proposed. The simulation results show that it can complete more event monitoring and prolong the lifetime of WSNs.

The rest of this paper is organized as follows: Section 2 reviews the related work. Section 3 introduces the system model and problem formulation. Section 4 introduces the dynamic clustering algorithm. Section 5 discusses the inter-cluster task assignment scheme. Section 6 introduces the intra-cluster one-to-multiple cooperative computing algorithm. Section 7 describes the simulation method and results. Section 8 is about the conclusion of our work.

## 2. Related Works

In this section, we first review relevant work about event-driven WSNs, dynamic clustering, edge computing and cooperative computing in WSNs. Then, we analyze the limitations of existing works and introduce the motivation of this paper.

### 2.1. Event-Driven WSNs

The basic functions of event-driven WSNs include event detection, data transmission, and result analysis [9]. First of all, it is fundamental to detect and identify events accurately. Niu et al. proposed a distributed detection scheme in the dense deployment of WSNs. The event is monitored by multi-node cooperation, and the fusion center makes judgments after collecting the data from each node [10]. Massive data transmission will cause channel congestion and excessive energy consumption. Some scholars have introduced quantitative measurement methods in the distributed detection, where the local nodes first quantify the measurements and then report them to the fusion center [11,12,13]. Some references focus on detecting events by local nodes without a fusion center. A local decision scheme based on multi-node cooperation was proposed in which events are identified by synthesizing multiple attributes of events [14].

For data transmission and result analysis, most of the existing literature focuses more on cloud-assisted solutions, and the common approach is to optimize network resource scheduling to minimize energy consumption. Wen et al. minimized the energy consumption of cloud-assisted application execution by optimizing the clock frequency configuration and data transmission scheme [15]. In [16,17], the authors achieved multi-hop data transmission with the lowest energy consumption by unified scheduling of sensor nodes through a centralized SDN server. In recent years, with the development of Unmanned Aerial Vehicle (UAV) technology, some scholars take UAVs as the computing offloading center to assist WSN to complete event monitoring [18].

In summary, the existing researches on event-driven WSNs paid more attention to the local detection of emergency events, multi-hop data return and cloud center processing. However, the above researches all rely on the cloud center to assist in the computing, so the round-trip transmission of data (especially massive data) will cause high delay and energy consumption. This is not an ideal approach for energy-constrained WSNs.

### 2.2. Dynamic Clustering

Clustering is proven to be an effective topology control and energy efficiency management mechanism in WSNs. The Low Energy Adaptive Clustering Hierarchy (LEACH) protocol [19] is one of the most well-known WSN clustering protocols, which rotates the cluster head among sensor nodes according to predetermined probability to avoid rapid depletion of cluster head energy. The Threshold-sensitive Energy-Efficient sensor Network protocol (TEEN) is a typical hierarchical clustering algorithm in event-driven WSNs [20]. TEEN can greatly reduce the amount of data transmission by setting hard and soft thresholds of event triggering. In order to further prolong the network lifetime, some location-aware protocols were proposed to reduce the transmission cost between nodes. The Hybrid Energy-Efficient Distributed clustering (HEED) protocol [21] takes into account the residual energy of nodes and the intra-cluster communication cost to make the cluster head distribution as uniform as possible. The EECS protocol proposed in [22] selects cluster heads according to the residual energy and location of nodes and forms a fair distribution of cluster heads. Considering the redundant data collected by the adjacent nodes, an enhanced clustering hierarchy approach was proposed to achieve energy efficiency in WSNs by using sleep and wake-up mechanism for overlapping and neighboring nodes [23].

In addition, *K*-means clustering algorithm is a typical distance-based clustering algorithm, which is very sensitive to the initial location of the cluster center. For each iteration, a different selection of the initial cluster center often leads to different clustering results [24]. Tanir et al. firstly selected the two points with the largest distance in the data set as the initial cluster center, allocated the remaining data objects to the corresponding cluster according to the distance to the cluster center point, and updated the cluster center [25].

However, it is difficult to control the size of clustering results in both classical clustering algorithms of WSNs and *K*-means algorithms. In this paper, we need the clustering results to be nearly the equal-size clusters in order to facilitate load balancing and subsequent algorithm execution.

### 2.3. Edge Computing and Cooperative Computing

Edge computing, a new computing mode, can migrate computation tasks or services from cloud centers to the edge of the network [26]. Computation offloading is the core concern of edge computing, which mainly includes three models: one-to-one offloading, multiple-to-one offloading, and one-to-multiple offloading. One-to-one offloading is a simple but classic model in which one terminal device offloads computation to one server [26]. Multiple-to-one offloading, in which multiple terminal devices offload computation tasks to a single server, is the main research model at present. A general approach is to design different heuristic offloading strategies through classical optimization theory [27,28,29]. However, it is difficult for the heuristic offloading algorithm to completely solve the optimization problem in the rapidly changing environment, especially for the Internet of Things with massive connections and dynamic access. Advances in artificial intelligence technology provide new ideas for solving such problems. For full task offloading, where computational tasks cannot be partitioned but can only be performed at terminal devices or at the edge servers, a DRL framework was proposed to minimize the trade-off between energy and latency [30]. For partial task offloading, where computation tasks can be further divided into multiple subtasks, two offloading algorithms based on Q learning and DDPG were proposed to minimize the execution delay of tasks [8]. One-to-multiple offloading is mainly used when the computing resources of servers are insufficient. Anselme et al. introduced the cooperation mechanism of edge servers, where edge servers located in the same cluster collaborate with each other. Within each cooperation space, a cooperative optimization problem was formulated to minimize a linear combination of bandwidth consumption and network latency [31]. In order to alleviate the burden on a single edge server and effectively utilize the computing resources of the whole edge network, Li et al. proposed a deep reinforcement learning algorithm to solve the complex computation offloading problem for the collaborative computing of heterogeneous edge servers [32].

The progress of MEMS technology has greatly improved the computing and storage capacity of terminal devices. Mining and scheduling the idle computing resources of terminal devices has become a new research hotspot. The paradigm of cooperative computing has been adopted by some scholars to study the computing offloading between devices. In [33], considering the system model in which the host device offloads data to multiple cooperative devices, Liu et al. designed the optimal offloading strategy by the convex optimization method to maximize the task load of the host device under the task delay constraint. In [34], a heuristic task allocation routing algorithm was proposed to fairly distribute computation tasks among multiple cooperative nodes. Wang et al. adopted game theory to realize the efficient allocation of computation tasks among multiple idle devices and edge servers [35]. However, the above references mainly focus on the computing resources scheduling among several rechargeable devices, such as mobile phones, and the network topology is generally static.

In WSNs, there are a few references for edge computing and cooperative computing. In order to deal with different types of delay-sensitive tasks, Xu et al. proposed a software-defined mission-critical wireless sensor network, in which tasks can be computed locally or offloaded to edge servers based on latency constraints [36]. In order to further explore the computing power of peer nodes, some pioneers have worked on cooperative computing in WSNs. For one-to-one partial task offloading, Sheng et al. built a one-to-one cooperative computing model and proposed a new method to minimize the energy consumption of a pair of nodes under delay constraint [3]. In [37], a collaborative computing framework for urgent task processing in WSNs was proposed and implemented on real devices. For high-density WSNs, Jiang et al. proposed an offloading algorithm to minimize network energy consumption while requested bandwidths and delay are satisfied [38]. However, in the above references, the computing services between nodes are one-to-one computation offloading. To the best of our knowledge, there is no literature about the fact that multiple peer nodes synchronously provide parallel computing services for one node in WSNs.

### 2.4. Motivation

To sum up, with the substantial improvement in the computing capacity of sensors, it has become possible to utilize local computing resources in WSNs to complete emergency event processing. However, it also brings new challenges to WSNs, and the existing literature has certain limitations in addressing these new challenges. Firstly, the algorithms designed in the above literature are mainly based on the cloud computing scenario, and the transmission energy consumption is the key concern. There is still a lack of system models that rely on network local computing resources to complete emergency event handling. Second, in order to perform the more complex task computations locally, more computing resources of nodes in WSNs should be utilized. However, in the existing literature, cooperative computing in WSNs is mainly about one-to-one cooperation and rarely involves one-to-multiple cooperation. As a result, WSN can only handle lightweight tasks due to insufficient computing capacity, so we try to introduce the one-to-multiple cooperation model in large-scale WSNs to activate the computing capacities of redundant nodes. Third, there are challenges to designing a computation offloading strategy for one-to-multiple cooperative computing in large-scale event-driven WSNs, such as the scheduling of a large number of nodes, an event-driven dynamic environment, and energy consumption balance in the whole network.

In order to overcome the above new challenges, in this paper, aiming at maximizing the number of completed events while extending the lifetime of the WSN, we first built an event-driven WSN model for handling events locally with one-to-multiple cooperative computing. Then, an equal-size *K*-means clustering algorithm and inter-cluster task assignment algorithm are proposed, which solve large-scale node management and task allocation problems among clusters. Next, in order to make each cluster efficiently complete the computation tasks within the deadline, a DDPG-based intra-cluster one-to-multiple cooperative computing algorithm is proposed to obtain a computation offloading strategy.

## 3. System Model and Problem Formulation

In this section, we first introduce the whole system model, including the large-scale event-driven WSN model, the event model, the network workflow, the energy model and the delay model. Next, we formulate and analyze the optimization problem.

### 3.1. The Large-Scale Event-Driven WSN Model

**Figure 1:**
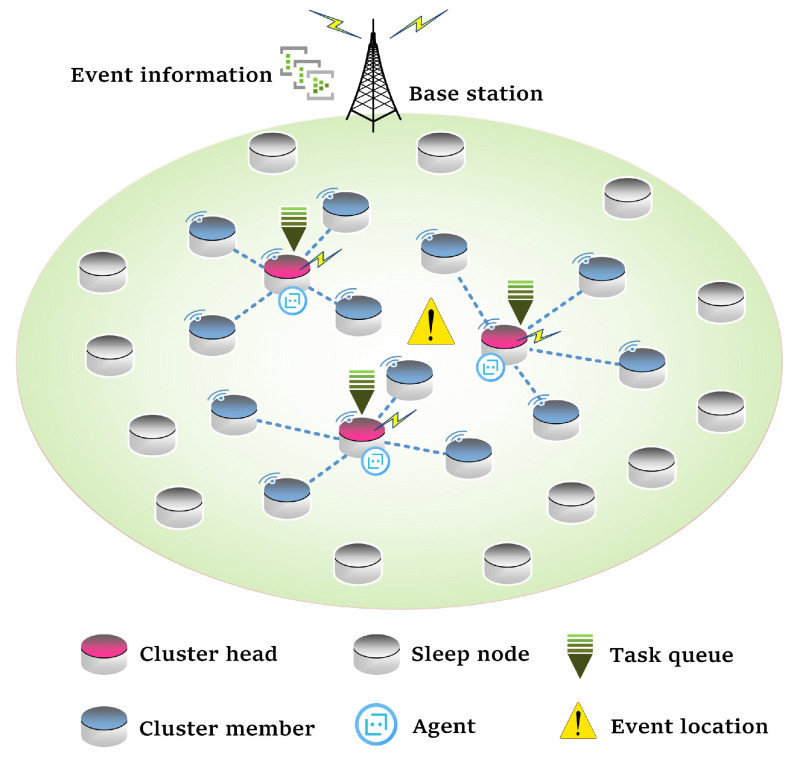
Event-driven WSN model.

In this paper, we consider that a large number of sensor nodes are randomly and densely distributed in the monitored area, and they are immovable after deployment, as shown in Figure 1. All nodes are of the same type, with the same computing capacity and initial battery energy, and the battery cannot be supplemented. The base station (BS) is fixed outside the monitored area and can directly communicate with all sensor nodes. All nodes can communicate with each other.

Random events occur successively in the monitored area. Unlike our previous studies, where only one node is activated and keeps monitoring the event. In this paper, the nodes that detect the event will be activated. Since the nodes are densely deployed, multiple nodes will be activated simultaneously ( When the event occurs within the node sense radius $R_{sense}$ and the monitored data exceed the preset threshold, the node is activated. A larger $R_{sense}$ means more nodes may be activated.). The active nodes are dynamically divided into several clusters by the clustering algorithm pre-installed in the BS. The cluster head continuously collects event monitoring data and sends monitoring reports (not raw data) to the BS. Cluster members can participate in cooperative computing but do not need to collect data. The BS generates a series of computation tasks according to monitoring reports and assigns them to the cluster heads. Each cluster head is regarded as an agent that performs the DRL algorithm to complete its own computation tasks locally through intra-cluster cooperative computing among cluster members.

### 3.2. Event Model

- Event-triggered. Monitoring and handling static events is the focus of this article, such as fire monitoring. To judge the event-trigger, local decision [39,40] or fusion center decision based on quantization [11,12] are viable. We use the set Z={1,2,3,…,Z} to denote all triggered events.
- Event composition. In the process of event monitoring, the corresponding applications are started, such as data preprocessing and actuator emergency start, which need to complete a series of computation tasks. We can describe an emergency event as a queue of computation tasks. The computation task queue of the *z*-th event can be expressed as $N_{z}=\left\{1,2,3,\ldots ,CT_{z}\right\}$. In this paper, we still consider a task model for partial offloading [26], which means that a computation task can be further divided into multiple subtasks. In addition, the time of event monitoring is divided into several uneven time slots and one task is processed in each time slot.
- Computation tasks. Each computation task in $N_{z}$ is completed locally by a cluster. Assume that a cluster has 1 + *N* nodes, including 1 cluster head and *N* cluster members. We define the set N={0,1,2,…,N} as the whole cluster and $N_{member}=\left\{1,2,\ldots ,N\right\}$ as cluster members, so the number of nodes in N and $N_{member}$ are (*N* + 1) and *N*. A canonical model is introduced to describe a computation task by $ct_{z}=\left(L_{ct_{z}},T_{deadline}^{ct_{z}}\right)$ [41], where $L_{ct_{z}}$ indicates the data size of the computation task that can be partitioned into several parts: $\left\{l_{0},l_{1},\ldots ,l_{N}\right\}$, where $l_{0}$ offloads to the cluster head and the others offload to cluster members. We define $\left\{l_{n}=a_{n}\times L|\sum_{i=0}^{N}a_{n}=1\right\}$, where $a_{n}$ represents the proportion of computation task $ct_{z}$ undertaken by the *n*-th cluster node. $T_{deadline}^{ct_{z}}$ indicates the task completion deadline.

### 3.3. The Workflow of Large-Scale Event-Driven WSN

**Figure 2:**
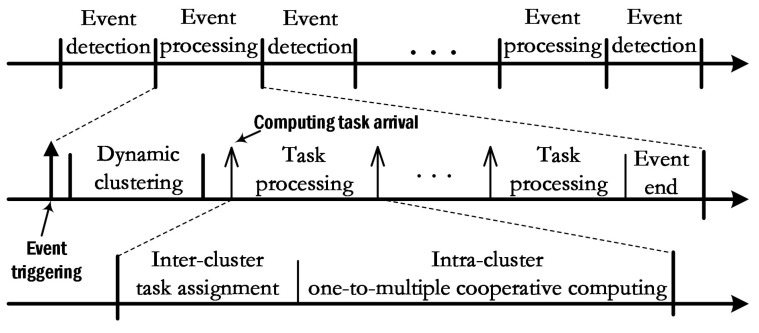
The workflow of event-driven WSN.

The network workflow involves two phases: event detection and event processing, as shown in Figure 2. During event detection, all nodes are in sleep mode and only the detection function opens. When the *z*-th event occurs, the nodes which detect the *z*-th event are activated and divided into several clusters according to the dynamic clustering algorithm pre-installed in BS. Only one clustering operation is performed for each event, which means that the topology of the WSN does not change during the process of monitoring *z*-th event. The cluster heads collect the data and send monitoring reports to the BS. Based on the monitoring reports and the energy and computing capacity of clusters, the BS assigns the computation tasks among the cluster heads ( in this process of the inter-cluster task assignment, the base station only sends the control commands, and the raw data are still stored in the cluster heads). Then, each cluster head performs the intra-cluster one-to-multiple cooperative computing algorithm, in which computation tasks are further divided into several subtasks $\left\{l_{n}|n\in N\right\}$ and offloaded to the cluster members. Cluster members return the calculation results of their subtasks to the cluster head. Then, the cluster head combines the local computing result and the cooperative computing results into the final result of the task $ct_{z}$ and returns to the BS.

### 3.4. Energy Model

In this paper, we mainly consider two parts of energy consumption in intra-cluster cooperation: computing energy consumption and transmission energy consumption. We assume that the delay and energy consumption for returning results to the cluster head is negligible [3]. In addition, since the data that are transmitted between the cluster head and the BS are the monitoring reports and commands, which are of a small number of bits, we ignore the transmission energy consumption between the cluster head and the BS.

(1) Computing energy model. The node CPU architecture adopts advanced Dynamic Voltage and Frequency Scaling (DVFS) technology. Thus, the energy consumption of *l*-bit computing tasks can be obtained by the following formula [33].

(1) $$E_{c}\left(l\right)=\kappa f^{3}t=\kappa f^{2}\omega l$$

where κ is the coefficient determined by the physical characteristics of a node’s CPU, *f* is the CPU frequency, *l* is the data size of a subtask, and *t* is the execution time of completing the *l*-bit task, t=ωl/f, ω represents the number of CPU cycles required to compute 1-bit data.

(2) Transmission energy model. We refer to the classical transmission energy consumption model in WSNs [19]. The energy consumption of transmitting *l*-bit data between two nodes can be expressed:

(2) $$E_{t}\left(l,d\right)=E_{elec}\cdot l+\xi_{amp}\cdot l\cdot d^{2}$$

where *d* is the distance between two nodes. The energy consumption of receiving this *l*-bit data is:

(3) $$E_{r}\left(l\right)=E_{elec}\cdot l$$

where $E_{elec}$ is the circuit energy consumption factor, which denotes the energy consumption of encoding and modulating 1-bit data. $\xi_{amp}$ is the amplifier energy consumption factor related to transmission loss.

### 3.5. Delay Model

Similar to the energy model, we mainly consider the delay of intra-cluster cooperation and ignore the transmission delay between the cluster head and the BS. The execution delay of the task ${ct}_{z}$ is related to the execution delay of subtasks $\left\{l_{n}|n\in N\right\}$, where $l_{0}$ is computed locally by the cluster head and $\left\{l_{n}|n\in N_{member}\right\}$ are computed by cluster members through remote cooperation. Therefore, the delay of the cluster head depends only on computing delay, while the delay of the cluster members includes the computing delay and communication delay. For communication delay, we mainly consider data transmission delay, ignoring propagation delay and the delay of returning results. At the same time, we assume that the communication channel is ideal, and *C* is the transmission rate. $\left\{a_{n}|n\in N\right\}$ represents the proportion of task offloading; specifically, $a_{0}$ is about the task proportion undertaken by the cluster head.

For the execution delay of the cluster head $t_{0}$:

(4) $$t_{0}=\frac{\omega l_{0}}{f_{0}}=a_{0}\cdot \frac{\omega L}{f_{0}}$$

where $l_{0}=a_{0}L$, and ω represents the number of CPU cycles required to compute 1 bit of data. $f_{0}$ is the CPU frequency of the cluster head.

For the execution delay of cluster members $t_{n}$, including the computing delay $t_{n}^{c}$ and the transmission delay $t_{n}^{t}$:

(5) $$t_{n}^{c}=\frac{\omega l_{n}}{f_{n}}=a_{n}\cdot \frac{\omega L}{f_{n}},\;n\in N_{member}$$

(6) $$t_{n}^{t}=\frac{l_{n}}{C}=a_{n}\cdot \frac{L}{C},\;n\in N_{member}$$

(7) $$t_{n}=t_{n}^{c}+t_{n}^{t},\;n\in N_{member}$$

Therefore, the execution delay of the task can be represented as

(8) $$t_{ct_{z}}=max\left(t_{n}|n\in N\right)$$

when $t_{ct_{z}}<T_{deadline}^{ct_{z}}$, the task is completed. Otherwise, it cannot be completed.

### 3.6. Problem Formulation

The core mission of event-driven WSNs is to successfully complete event monitoring. In this paper, an event consists of a series of computation tasks, so the completion rate of tasks is a key indicator to determine whether an event is completed. For a computation task $ct_{z}$, if $L_{ct_{z}}$-bit are computed within the deadline $T_{deadline}^{ct_{z}}$, the computation task is complete. For an event *z*, the definition of event monitoring completion is as follows: if the completion rate of computation tasks is more than 95%, the event is completed (This paper focuses on relying on local computing capacity to complete computation tasks. If cloud-assisted computing and error control mechanisms are adopted, the completion rate of computation tasks can be further improved). The number of completed events in Z is denoted by $U_{ce}$.

The object of this work is to maximize $U_{ce}$ before the WSN crashes by solving inter-cluster task assignments and intra-cluster multi-node cooperative computing problems. We formulate the optimization problem as follows:

(9) $$\begin{array}{c} \max_{\Pi ,\Omega ,l_{0},l_{1},\ldots ,l_{N}}\;U_{ce},\;ct\in N_{z},\;z\in Z\\ s.t.\;\left\{\begin{array}{l} c_{1:}\sum_{n=0}^{N}l_{n}=L,\;0\leq l_{n}\leq L\\ c_{2:}\sum_{n=0}^{N}a_{n}=1,\;0\leq a_{n}\leq 1\\ c_{3:}\;0\leq t_{ct}\leq T_{deadline}\\ c_{4:}\;E_{dead}\leq E_{n}\leq E_{init} \end{array}\right. \end{array}$$

where Π is the dynamic clustering policy of active nodes. Ω is inter-cluster task assignment policy. $l_{0},l_{1},\ldots ,l_{N}$ are the task partitions of $ct_{z}$, and *N* is the number of nodes in a cluster. $c_{1}$ ensures that the computation task is fully allocated and subtask $l_{n}$ does not exceed *L*. $c_{2}$ is similar to $c_{1}$, where $a_{n}$ refers to the offloading proportion of the computation task. $c_{3}$ represents the delay constraints of the task. $c_{4}$ represents the energy constraints of nodes, where $E_{n}$ denotes the remaining energy of the node, $E_{init}$ denotes the initial energy when nodes are deployed, and $E_{dead}$ denotes the remaining energy of nodes before death, which is 10% of $E_{init}$ in this paper. In [42], this kind of problem proved to be an NP-hard problem, and it is difficult to find an optimal solution.

We can analyze the problem from two aspects. First, based on the previous analysis, computing capacity is the main factor affecting the completion of events. The more urgent the task (the smaller $T_{deadline}^{ct_{z}}$), the more nodes that are needed to participate in cooperative computing to ensure the completion rate of the task. For example, if $L_{ct_{z}}$-bit of a task is divided into three parts $\left\{l_{0},l_{1},l_{2}\right\}$, which are completed by the cluster head and two cluster members, the task execution delay is shorter than that for the cluster head that undertakes the whole task. Therefore, the task completion can be beneficial by having more nodes involved in the computing. Second, energy consumption directly affects the number of completed events. On the one hand, since the sensor energy is non-renewable, low energy consumption can prolong the WSN lifetime. On the other hand, the cluster head is responsible for receiving and distributing tasks, and its survival is the guarantee of task completion.

However, there is a fact that can not be ignored in WSNs: transmission energy consumption is much higher than computing energy consumption [19], which means that the energy consumption caused by too much cooperative computing is enormous. An optimal scheme is to reduce the amount of cooperative computing as little as possible under the condition of satisfying the delay requirement. Therefore, we decompose the original problem into three subproblems. In Section 4, we discuss dynamic clustering of active nodes, which is the basis of the subsequent algorithm. In Section 5, we discuss inter-cluster task assignment, which helps to realize task balancing among different clusters. In Section 6, we propose an intra-cluster one-to-multiple cooperative computing algorithm based on DRL to minimize the total energy consumption of cluster nodes under delay constraints.

## 4. Dynamic Clustering of Active Nodes

Due to the dense deployment of nodes, a large number of nodes are activated by event triggering. Considering the following factors, we need to cluster the active nodes. Firstly, clustering is beneficial to the efficient utilization of node computing capacity and the balance of energy consumption. Secondly, nodes take turns acting as cluster heads, which can effectively avoid task failure caused by cluster head death. Third, too many cluster members will bring dimensional disaster to cooperative computing decisions and significantly increase the computational complexity of the algorithm.

For the clustering results, we consider that it should be uniform, that is, the size of every cluster is basically the same. This is not only beneficial to energy consumption balancing, but also to the implementation of subsequent algorithms. Then, cluster members should keep a short distance to facilitate communication, and the cluster head should have sufficient energy. Therefore, we propose an equal-size *K*-means clustering algorithm to achieve the above goals.

*K*-means is a widely used clustering algorithm. The main idea is: given the *K* value and *K* initial cluster centers, each element is assigned to the cluster represented by the nearest cluster center. However, the traditional *K*-means algorithm can not achieve uniform clustering, so it is improved by our algorithm. Firstly, all nodes are roughly clustered according to the distance of nodes, and then every cluster is adjusted one by one to make the size of each cluster equal. It is mainly divided into four stages as follows. See Algorithm 1 for the pseudo-code of dynamic clustering.
**Algorithm 1** Dynamic clustering algorithm. 1:Initialize *M* and *N* 2:Initialize *K* according to (10) 3:Initialize the position of $\left\{v_{k}\right\}$ according to (11) 4:Perform primary clustering and obtain $\left\{c_{v_{k}}\right\}$ 5:Initialize $C_{temp}=\left\{c_{v_{k}}\right\},C_{final}=Ø$ 6:**while** total node number of $C_{final}\neq M$ **do** 7:    find $c_{min}$ in $C_{temp}$ 8:    calculate the node number $M_{temp}$ in $C_{temp}$ 9:    **if** $\delta_{M_{temp},N}\neq 0$ **then**10:        **if** $N_{c_{min}}+1>\delta_{M_{temp},N}$ **then**11:           go to line 1812:        **else**13:           $c_{min}$ member updates until $N_{c_{min}}+1=\delta_{M_{temp},N}$14:        **end if**15:    **else**16:        $c_{min}$ member updates until $N_{c_{min}}+1=N+1$17:    **end if**18:    remove $c_{min}$ from $C_{temp}$19:    add $c_{min}$ to $C_{final}$20:**end while**

### 4.1. Initialization

In this stage, the *K* value (*K* denotes the number of clusters) and positions of *K* cluster centers need to be initialized, which have a great impact on the clustering effect.

Initialize *K* value with the following formula:

(10) $$\begin{array}{c} \left\{\begin{array}{c} K=\frac{M}{N+1}\;\delta_{M,N}=0\\ K=\frac{M}{N+1}+1\;\delta_{M,N}\neq 0\\ \delta_{M,N}=M\;\mathrm{mod}\;\left(N+1\right) \end{array}\right. \end{array}$$

where *M* denotes the number of active nodes, which is related to node sensing radius $R_{sense}$. *N* denotes the number of cluster members. *N* determines the computing capacity of the clusters, which can be adjusted according to the urgent situation of the task and will also affect the complexity of the subsequent algorithm. $\delta_{M,N}$ is the remainder of *M* over *N* + 1.

**Figure 3:**
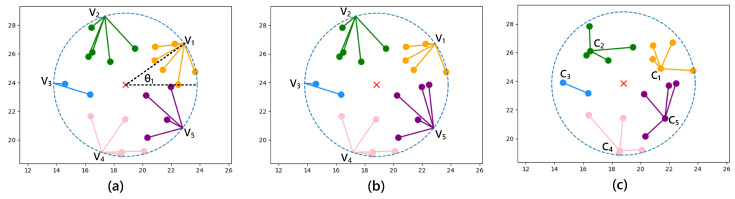
Dynamic clustering, (**a**) Initialization and promary clustering; (**b**) Fine adjustment; (**c**) Cluster head selection.

For initial positions of *K* cluster centers, different from the classical *K*-means algorithm, which randomly selects the initial cluster center, we evenly distribute the location of the initial cluster center $\left\{v_{k}|1\leq k\leq K\right\}$ on the edge of the event monitored area ( the edge refers the circle with the location of the event as the center and $R_{sense}$ as the radius). This can maximize the distance between the initial cluster centers and facilitate better results in primary clustering. The position coordinates of $\left\{v_{k}|1\leq k\leq K\right\}$ are obtained according to the following formula:

(11) $$\begin{array}{c} \left\{\begin{array}{c} x_{v_{k}}=x_{event}+R_{sense}\cdot cos\theta_{k}\\ y_{v_{k}}=y_{event}+R_{sense}\cdot sin\theta_{k}\\ \theta_{k+1}=\theta_{k}+2\pi /K,\;1\leq k\leq K \end{array}\right. \end{array}$$

where $\left(x_{v_{k}},y_{v_{k}}\right)$ denote the position coordinates of initial cluster center $\left\{v_{k}|1\leq k\leq K\right\}$, $\left(x_{event},y_{event}\right)$ denotes the position coordinates of the event, $\theta_{k}$ denotes the angle between the position vector $\left(x_{v_{k}}-x_{event},y_{v_{k}}-y_{event}\right)$ and the X-axis, $\theta_{1}$ is a random value from 0 to 2π. An example of initialization is shown in Figure 3a. The red cross indicates where the event occurred. Within the monitoring range of the event, *M* = 22 nodes are activated. We choose the number of cluster members *N* = 4, then $\delta_{M,N}$ = 2, so *K* = 5 according to (10). Next, according to (11), five initial cluster centers $\left\{V_{1},V_{2},V_{3},V_{4},V_{5}\right\}$ are uniformly initialized at the edge of the monitored area.

### 4.2. Primary Clustering

*M* active nodes successively calculate the distance to the initial cluster center $\left\{v_{k}|1\leq k\leq K\right\}$, and then join the cluster with the nearest cluster center. After primary clustering, the nodes with similar distances can be basically divided into a cluster, but the number of nodes in each cluster will be different. We denote these *K* clusters as the set $\left\{c_{v_{k}}|1\leq k\leq K\right\}$. As shown in Figure 3a, the number of nodes in $\left\{c_{v_{1}},c_{v_{2}},c_{v_{3}},c_{v_{4}},c_{v_{5}}\right\}$ is 6, 5, 2, 5, 4, respectively.

### 4.3. Fine Adjustment

The basic idea of fine adjustment is that a small-size cluster, in turn, selects a node from a large-size cluster, and the selected node has the shortest distance from the small-size cluster (see lines 5–16 in Algorithm 1). First, two sets, $C_{temp}$ and $C_{final}$, need to be initialized. In initial status, all $\left\{c_{v_{k}}|1\leq k\leq K\right\}$ are stored in $C_{temp}$ while $C_{final}$ is empty. Starting from cluster $c_{min}$, which has the least number of nodes in $C_{temp}$, we adjust the number of nodes in each cluster one by one. The adjusted clusters are added to $C_{final}$ and removed from $C_{temp}$ until *M* active nodes all in $C_{final}$. In line 8 and 9, $M_{temp}$ denotes the number of current nodes in $C_{temp}$, and $\delta_{M_{temp},N}=M_{temp}\;\mathrm{mod}\;\left(N+1\right)$. The function of the algorithm in line 9–11 is to form a cluster with $\delta_{M_{temp},N}$ nodes, and then adjust the size of the remaining clusters to (*N* + 1). The method of member updating in line 10 and 12 involves, from all the nodes in $C_{temp}$, finding the node closest to the center $v_{c_{min}}$ of the cluster $c_{min}$ and shifting it into cluster $c_{min}$.

Returning to the previous example, Figure 3a shows the result of primary clustering. At this time, $C_{temp}=\left\{c_{v_{1}},c_{v_{2}},c_{v_{3}},c_{v_{4}},c_{v_{5}}\right\}$, so $M_{temp}$ = 22 and $\delta_{M_{temp},N}$ = 2. In the first iteration, $c_{3}$ with only two nodes is selected as $c_{min}$. Since ($N_{c_{v_{3}}}$  + 1) = $\delta_{M_{temp},N}$ = 2, the member update is not performed, so $c_{3}$ is moved from $C_{temp}$ to $C_{final}$. In the second iteration, $C_{temp}=\left\{c_{v_{1}},c_{v_{2}},c_{v_{4}},c_{v_{5}}\right\}$, so $M_{temp}$ = 20 and $\delta_{M_{temp},N}$ = 0. $c_{v_{5}}$ with four nodes is selected as $c_{min}$ since $N_{c_{v_{5}}}+1=4<5$, and the member update is performed. Shift a node from cluster $c_{v_{1}}$ to cluster $c_{v_{5}}$, as shown in Figure 3b. Then, the node number of cluster $c_{v_{5}}$ is 5, so the member update is not required, and cluster $c_{v_{5}}$ is moved from $C_{temp}$ to $C_{final}$. In the next three iterations, $c_{v_{1}}$, $c_{v_{2}}$ and $c_{v_{4}}$ are, respectively, taken as $c_{min}$. Since the node number of these three clusters is all 5, they are directly moved to $C_{final}$ without the member update. At the end, $C_{final}$ = $\left\{c_{v_{3}},c_{v_{5}},c_{v_{1}},c_{v_{2}},c_{v_{4}}\right\}$, and corresponding number of nodes is {2, 5, 5, 5, 5}.

### 4.4. Cluster Head Selection

Next, we need to select a cluster head in each cluster. Since cluster heads are the core of receiving and distributing tasks, their energy consumption is relatively huge, and the survival of cluster heads is the guarantee of task completion. Therefore, the node with the largest residual energy in the cluster is selected as the cluster head, and we define the cluster headset of the *z*-th event $C_{z}=\left\{c_{k}|1\leq k\leq K\right\}$, as shown in Figure 3c.

## 5. Inter-Cluster Task Assignment

During the process of monitoring the event *z* for each task $ct_{z}$ in $N_{z}$, the BS needs to assign the task $ct_{z}$ to an optimal cluster. Since some nodes may die during the execution of the task, the computing capacities and residual energy of nodes in each cluster are constantly changing. The cluster with more residual energy and higher computing capacity is more suitable for performing the current computation task. Therefore, the selection of the optimal cluster should be the trade-off between energy and computing capacity. We introduce the parameters λ (0≤λ≤1) to measure the weight of the residual energy of clusters and (1−λ) to measure the weight of the computing capacity of clusters. The formula of the optimal cluster opt_c selection is as follows:

(12) $$opt\_c=argmax(\lambda \cdot E_{c_{k}}+\left(1-\lambda \right)\cdot f_{c_{k}}),\;1\leq k\leq K$$

where $E_{c_{k}}$ denotes the sum of the remaining energy of all nodes in the cluster $c_{k}$, and $f_{c_{k}}$ denotes the sum of CPU frequencies of all nodes in the cluster $c_{k}$.

## 6. Intra-Cluster Multi-Node Cooperative Computing

In this section, we discuss the intra-cluster one-to-multiple cooperative computing problem. Although there are many clusters involved in task computing, in fact, each cluster faces a similar state. We only need to discuss the problem of multi-node cooperation in a single cluster, and we extend the designed algorithm to other clusters. First, intra-cluster one-to-multiple cooperative computing problem is abstracted as Markov Decision Processes (MDPs). Then, we propose a DDPG algorithm to solve the optimization problem of offloading actions. The DDPG algorithm contains four neural networks and an experience pool. Although the model training is performed offline in a single cluster, the model inference and testing is on the whole network with the trained model deployed in each cluster. We also give the corresponding model inference method at the end.

### 6.1. Markov Decision Process

In a cluster, the cluster head can be considered an agent, and its offloading decision problem can be modeled as an MDP problem. The MDP problem consists of three parts: state space *S*, action space *A*, reward function *r*. In a cluster, performing a computation task is regarded as a step, and each step consists of an observing state, performing an action, state transition and obtaining a reward.

(1) State space *S*: We define the state space

(13) $$S=\{S_{node},S_{task}\}$$

where the $S_{node}$ describes the states of all nodes in the cluster, $S_{task}$ describes the state of the current computation task processed by the cluster.

Specifically, $S_{node}=\left\{S_{node_{n}}|0\leq n\leq N\right\}$, where *N* denotes the number of cluster members, $S_{node_{0}}$ denotes cluster head state and $\left\{S_{node_{n}}|1\leq n\leq N\right\}$ denotes the cluster member states. For each $S_{node_{n}}$, it has three components $\left\{S_{n}^{E},S_{n}^{f},d_{n}^{0}\right\}$: $S_{n}^{E}$ denotes the remaining energy of node *n*, $S_{n}^{f}$ denotes the CPU frequency of node *n*, $d_{n}^{0}$ denotes the distance between cluster head and node *n*.

$S_{task}$ has two components $\left\{L_{ct_{z}},T_{deadline}^{ct_{z}}\right\}$, $L_{ct_{z}}$ indicates the data size of the computation task that can be partitioned into several parts $\left\{l_{n}|0\leq n\leq N\right\}$, $T_{deadline}^{ct_{z}}$ denotes the task completion deadline.

(2) Action space *A*. When the cluster head receives a computation task, it must make an action to assign the task to each node for processing. We define the action space

(14) $$A_{ct_{z}}=\left\{a_{ct_{z}}^{0},a_{ct_{z}}^{1},\ldots ,a_{ct_{z}}^{N}\right\},\;0\leq a_{ct_{z}}^{n}\leq 1$$

each element $a_{ct_{z}}^{n}$ denotes the proportion of the computation task offloading to the *n*-th node, and the constraint is:

(15) $$a_{ct_{z}}^{0}+a_{ct_{z}}^{1}+\ldots +a_{ct_{z}}^{N}=1$$

(3) Reward function *r*. The object of this paper is to maximize the number of completed events. From the previous analysis, the key to achieving this object is to complete more computation tasks with less energy consumption under the delay constraint. Therefore, the reward function is the trade-off between energy consumption and delay, and the formula is as follows:

(16) $$r=\mu \cdot r_{t}+\nu \cdot r_{e}$$

where μ,ν are weight parameters, which need to be adjusted during training. $r_{t}$ and $r_{e}$ are related to delay and energy consumption of a single task, respectively:

(17) $$\begin{array}{c} r_{t}=\left\{\begin{array}{c} 1,\;t_{ct_{z}}\leq T_{deadline}^{ct_{z}}\\ -0.5,\;t_{ct_{z}}>T_{deadline}^{ct_{z}} \end{array}\right. \end{array}$$

where $t_{ct_{z}}$ is the task execution time, which is obtained from Equation (8). When the task is not completed within the deadline, a negative reward is given, and when the task is completed within the deadline, a high positive reward is given.

(18) $$r_{e}=-\left(\sum_{n=0}^{N}E_{c}\left(l_{n}\right)+\sum_{n=1}^{N}E_{t}\left(l_{n},d_{n}^{0}\right)+\sum_{n=1}^{N}E_{r}\left(l_{n}\right)\right)$$

where $l_{n}=a_{n}L$ denotes the size of subtasks undertaken by node *n*. $E_{c}\left(l_{n}\right),E_{t}\left(l_{n},d_{n}^{0}\right),E_{r}\left(l_{n}\right)$, respectively, denote the computation energy consumption, transmission energy consumption, and reception energy consumption, which can be obtained by Equations (1)–(3). When the total energy consumption of the cluster to execute a task is larger, a more negative reward will be given.

For every task, an agent makes an action, and the environment automatically gives a reward. The total reward is $\sum_{ct_{z}=1}^{CT}r_{ct_{z}}$. The goal of our DRL algorithm is to maximize the total reward.

### 6.2. Design and Training for DDPG-Based One-to-Multiple Cooperative Computing

Classical reinforcement learning algorithms, such as Q-learning and DQN, can only handle discrete action space. For continuous action space, Q-learning and DQN can only approximate the action space by discretizing the action, which will affect the performance of the algorithm. In the comparative experiment of this paper, we also adopt the DQN algorithm to realize one-to-multiple cooperative computing through the predefined discrete offloading ratio.

**Figure 4:**
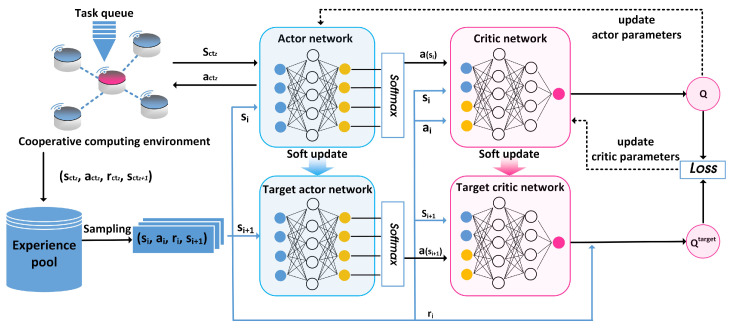
The training of the DDPG algorithm.

The DDPG algorithm extends the DRL action space to a continuous domain and adopts the actor–critic schema. Compared with the random strategy, it has only one action choice in each step, which is helpful in improving the convergence of training. The DDPG algorithm contains four deep neural networks: the actor network $\pi (s|\theta^{\pi })$ with the parameter represented by $\theta^{\pi }$, the critic network $Q(s,a|\theta^{Q})$ with the parameter represented by $\theta^{Q}$, the target actor network $\pi^{\prime }\left(s|\theta^{\pi^{\prime }}\right)$ with the parameter represented by $\theta^{\pi^{\prime }}$, and the target critic network $Q^{\prime }\left(s,a|\theta^{Q^{\prime }}\right)$ with the parameter represented by $\theta^{Q^{\prime }}$. The two networks of actors and the two networks of critics have the same structure, respectively. The algorithm framework is shown in Figure 4, and the pseudo-code of the algorithm is shown in Algorithm 2.
**Algorithm 2** Training of the DDPG algorithm. 1:Input the number of cluster member *N*. 2:Randomly initialize actor network parameter $\theta^{\pi }$ and critic network parameter $\theta^{Q}$. 3:Let $\theta^{\pi^{\prime }}\leftarrow \theta^{\pi }$ and $\theta^{Q^{\prime }}\leftarrow \theta^{Q}$ 4:**for** *episode* in range(Max_Iteration) **do** 5:    Initialize node state and task queue. 6:    **while** $E_{0}\geq E_{dead}$ **do** 7:        Obtain the current state $s_{ct_{z}}$ from environment. 8:        Select the offloading action $a_{ct_{z}}$ with (20). 9:        Obtain reward $r_{ct_{z}}$ with (16) and next state $s_{ct_{z}+1}$.10:        Store the record $\left(s_{ct_{z}},a_{ct_{z}},r_{ct_{z}},s_{ct_{z}+1}\right)$ to the experience pool.11:        Randomly sample *B* records.12:        Compute the target *Q* value with (19).13:        Update the parameters of critic network by minimizing the loss function (22).14:        Update the parameters of actor network by policy gradient with (23).15:        Update two target network with (24).16:    **end while**17:**end for**

The actor network is responsible for selecting actions based on states to perform computation offloading. Then, a record about $\left(s_{ct_{z}},a_{ct_{z}},r_{ct_{z}},s_{ct_{z}+1}\right)$ is stored in the experience pool. The target critic network and the target actor network are responsible for evaluating the target *Q* value by utilizing the experience pool, and the critic network is responsible for calculating the *Q* value. The calculation method of the target *Q* value is given:

(19) $$Q_{ct_{z}}^{target}=r_{ct_{z}}+\gamma Q^{\prime }\left(s_{ct_{z}+1},\;\pi^{\prime }\left(s_{ct_{z}+1}|\theta^{\pi^{\prime }}\right)|\theta^{Q^{\prime }}\right)$$

where $r_{ct_{z}}$ represents the reward after executing the $ct_{z}$-th task, $s_{ct_{z}+1}$ represents the state when agent receives the task that is next to the $ct_{z}$-th task, and γ∈[0,1] denotes discount factor, which indicates the trade-off between the future and the current reward.

In order to increase the randomness of the training process and better explore the whole solution space, we add noise η in the learning process. At the same time, to satisfy the constant of Equation (15), we add a Softmax function. Through the Softmax function, the action value output by the actor network can be converted into a probability distribution whose range is [0, 1] and the sum is 1. The action selection expression is defined as follows:

(20) $$A_{ct_{z}}=Softmax\;\left(\pi \left(s|\theta^{\pi }\right)+\eta \right)$$

The Softmax function is defined as follows (taking the action expression of *n*-th node as an example):

(21) $$Softmax\left(a_{ct_{z}}^{n}\right)=\frac{e^{a_{ct_{z}}^{n}}}{\sum_{i=0}^{N}a_{ct_{z}}^{i}}$$

When updating the parameters of the critic network, the classical gradient descent algorithm is used to minimize the difference between the target *Q* value and the output of the critic network. The loss function is a squared loss function. The formulation of the loss function is given:

(22) $$Loss=\frac{1}{B}\sum_{i=1}^{B}{\left(Q_{i}^{target}-Q\left(s_{i},a_{i}|\theta^{Q}\right)\right)}^{2}$$

where *B* denotes the size of a batch. We adopt the Adam optimizer to minimize the loss function, and the policy gradient of the actor network is defined as follows:

(23) $${▽}_{\theta^{\pi }}J\approx \frac{1}{B}\sum_{i}{▽}_{a}Q\left(s,a|\theta^{Q}\right){{|}_{s=s_{i},a=\pi \left(s_{i}\right)}{▽}_{\theta^{\pi }}\pi \left(s|\theta^{\pi }\right)|}_{s_{i}}$$

For the target network parameter update, different from DQN, which directly copies the corresponding parameters, the DDPG algorithm uses a gradual update method that the actor network and the critical network to perform soft target updates on the target network. The specific way is as follows:

(24) $$\begin{array}{c} \theta^{Q^{\prime }}\leftarrow \tau \theta^{Q}+\left(1-\tau \right)\theta^{Q^{\prime }}\\ \theta^{\pi^{\prime }}\leftarrow \tau \theta^{\pi }+\left(1-\tau \right)\theta^{\pi^{\prime }} \end{array}$$

where 0<τ<1 denotes the soft update speed of the target network.

For environment parameters, we first need to determine the number of cluster members *N*, because it affects the dimension of state *S* and action *A*. The initial value of *N* is determined by the clustering algorithm but the node death will cause a reduction in the *N* value of each cluster during the execution of the task, which leads to the failure of the algorithm based on the initial *N* value. To solve this problem, we need to train different action networks $\left\{\pi_{n}|1\leq n\leq N\right\}$ for different values of *N*. For example, if the initial value of *N* is 4, we need to execute the DDPG algorithm four times in order to train four actor networks $\pi_{1},\pi_{2},\pi_{3},\pi_{4}$, corresponding to *N* equals 1, 2, 3, 4, respectively.

For the neural network, the dimension of the input layer of the actor network is 3(*N* + 1) + 2, which describes the node state and task state in Equation (13), and the dimension of the output layer of the actor network is (*N* + 1). The input layer of critic network includes two parts, the state *S* and the action *A*. The dimension of the output layer of the critic network is 1, representing the *Q* value. The hidden layer of the actor and critic network consists of fully connected layers. Meanwhile, we use layer normalization technology to standardize the input data in each neural network. The sigmoid function is used as the activation function for each neural network.

At the beginning of the training, the decision made by the agent is close to a random algorithm. With the continuous iteration of the algorithm, the reward for the offloading policy gradually rises and finally converges to the near-optimal algorithm. At the end of the learning iteration, the learned parameters of the DDPG neural network are obtained.

### 6.3. Inference for DDPG-Based One-to-Multiple Cooperative Computing

The neural model parameters are obtained by training in a single cluster environment, which can not fully test the performance of the model, so we need to infer and test the model in the whole WSN environment. The pseudo-code is shown in Algorithm 3.
**Algorithm 3** Inference of DDPG algorithm. 1:Initialize node distribution, node energy, event_set Z. 2:Import the trained actor networks $\left\{\pi_{n}|1\leq n\leq N\right\}$. 3:**while** *SYSTEM_END* ≠ *True* **do** 4:    **for** *z* in Z **do** 5:        Check the remaining energy of nodes. 6:        Obtain active node set. 7:        Perform dynamic clustering algorithm, and get the clustering result $C_{z}$. 8:        **for** *task* $ct_{z}$ in $task\_set\;N_{z}$ **do** 9:           Find the optimal cluster opt_c with (12).10:           Cluster head of opt_c judges current *N* value.11:           Cluster head of opt_c loads the corresponding actor network parameters.12:           Perform cooperative computing based on the current state *S*.13:           Update the remaining energy of nodes.14:        **end for**15:    **end for**16:**end while**

After the initialization, we import *N* sets of neural network parameters that have been trained ( In actual deployment, the neural network architecture is preset in the node, and the parameters are stored in the node memory. The network model loads different parameters according to different *N* values.). The test period is the entire life cycle of the WSN ( when the number of dead nodes exceeds more than half of the total number, we consider that the WSN will not work normally due to coverage holes, so *SYSTEM_ END = True* at this time.). Random events occur successively, and the nodes around them are activated. The active node performs the clustering algorithm. For each task of the event, the optimal cluster is selected according to Equation (12). According to the current *N* value, the cluster head of the optimal cluster loads the corresponding actor network parameters and performs cooperative computing actions according to the current state.

### 6.4. Computational Complexity Analysis

The proposed DDPG algorithm is an offline learning and off-policy DRL-based algorithm, so the stages of training and inference are performed separately, and the training process will not bring an external computing burden to the inference. Once the trained network converges, the solution can be generated quickly with a few simple algebraic calculations [43].

In the training stage, we need to execute the DDPG algorithm *N* times, and four neural networks need to be trained simultaneously in each training in order to obtain *N* sets of actor network parameters. The value of *N* actually represents the computing capacity of the cluster. We should choose an appropriate *N* according to the task in the application scenario. For example, if the tasks of an event are mostly of many bits and short deadlines, which means more computing capacity is needed, a large value of *N* should be chosen to ensure the completion rate of tasks. For the simulation scenario of this paper, *N* = 4 is enough to complete the computation tasks ( In the actual engineering design, *N* can be set according to the needs of the project, but too large an *N* will also lead to the problem of high algorithm complexity and uneven energy consumption. For some tasks with a large number of bits and low latency, cloud-assisted methods can also be used, which is not the focus of this paper.).

In the inference stage, only an actor network is required, which will be loaded with different parameters based on *N* values to complete the offloading decision. For each task, the action of the cluster head is generated by running the actor network and the Softmax function once. Since the fully-connected hidden layer is adopted in the neural network of the proposed DDPG algorithm, the computational complexity of an operation performed by the actor network is $O(\sum_{c=1}^{C}n_{c}\cdot n_{c-1})$, where *C* is the number of network layers, $n_{c}$ is the number of neurons in the *c*-th layer [43], and the computational complexity of the Softmax function is O(N). Then, the computational complexity of generating an action of the cluster head is $O(\sum_{c=1}^{C}n_{c}\cdot n_{c-1}+N)$. Therefore, the overall complexity of the proposed DDPG algorithm is $O(\left(\sum_{c=1}^{C}n_{c}\cdot n_{c-1}+N\right)\cdot \sum_{z=1}^{Z}CT_{z})$, where *Z* is the number of events and $CT_{z}$ is the number of tasks of the *z*-th event. Moreover, we use a relatively shallow neural network (less than 5 layers), which has low computational complexity, and the existing embedded systems are fully competent for the inference work.

## 7. Performance Evaluation and Simulation Results

In this section, we evaluate the performance of DDPG-based one-to-multiple cooperative computing for large-scale event-driven WSNs. The simulations are carried out on the Python platform, where the Pytorch module is used to build the neural network model, and the Gym module is used to complete the environment construction.

### 7.1. Simulation Environment Setting

Since the training and inference of the algorithm are separated, the training is carried out in a single cluster, and the inference is carried out in the whole WSN. Therefore, we set up two environments for training and inference. In order to obtain better algorithm performance, the training environment should be similar to the inference environment. The initial *N* value is four in the simulation.

For the training environment, we need to simulate a single cluster cooperative computing environment and build a deep neural network. We randomly deploy (*N* + 1) nodes in the 20 × 20 m${}^{2}$ monitoring area and randomly select a cluster head from them. All nodes have the same initial energy and can communicate with each other. In a training episode, the cluster head successively receives computation tasks and makes offloading decisions until the cluster head energy is exhausted. The two task parameters $L_{ct_{z}}$ and $T_{deadline}^{ct_{z}}$ are also randomly set from their ranges. The value of the node sensing radius $R_{sense}$ is related to node density, which is discussed in the simulation results. The parameters used in the training environment are shown in Table 1, in which some parameter settings are referred to in the literature [3,33,37]. The hidden layer of DDPG’s actor networks and critic networks is a three-layer structure, and the number of neurons is 256, 128, 64, respectively. The setting of the reward parameters (μ,ν) in (16) has an influence on the convergence of the algorithm to the optimal value. These two parameters need to be manually adjusted and slightly changed according to different *N* values. We will discuss this in the simulation results. The other hyperparameters of neural networks are shown in Table 2.

For the inference environment, we simulate the full life cycle of the WSN. The the monitored area is 300×300 m${}^{2}$. A set of two-dimensional coordinates are randomly generated to represent the random deployment of nodes in the monitored area. Then, BS can obtain the node coordinates and notify every node. A communication channel is ideal so that the communication between nodes is stable and reliable. A randomly generated event queue demotes the event set Z, and each event contains two elements: event location and a set of computation tasks. Event location is a two-dimensional coordinate randomly generated within the monitored area. For each computation task, the setting methods of parameters $L_{ct_{z}}$ and $T_{deadline}^{ct_{z}}$ are the same as in the training environment. The nodes within the range centered on the event location with the radius of $R_{sense}$ are activated and divided into several clusters by the dynamic clustering algorithm. The parameter λ of inter-cluster task assignment in (12) is set as 0.5. The actor network structure is consistent with that in the training environment. The other parameters are the same as the training environment.

### 7.2. Comparative Algorithms

To better evaluate the performance of the algorithm, there are five other offloading methods compared with the proposed algorithm.

(1) DQN-based offloading algorithm: Since the DQN algorithm can only deal with discrete action space, we selected a set of representative actions for different values of *N*. For example, when *N* = 4, we have 100 sampling actions, such as (0.2, 0.2, 0.2, 0.2, 0.2), (0.6, 0.2, 0.1, 0.1, 0), and so on. Similar to the DDPG algorithm, DQN also obtains neural network parameters by training in a single cluster environment and then performs an inference test in the whole WSN.

(2) Exhaustive search: For each task, the cluster head searches all the actions with a step size of 0.01 and then calculates the delay and energy consumption after executing each action. The action with the least energy consumption is selected as the optimal solution with the constraint of delay. With the increase in *N*, the computational complexity of this algorithm will increase explosively.

(3) Local computing: The cluster head completes all the computation tasks and does not cooperate with the cluster members.

(4) Random offloading: The cluster head randomly selects an action in the action space and performs the corresponding computation offloading.

(5) Average offloading: The cluster head divides computation tasks into *N* + 1 parts on average, and the cluster head and each member all participate in computing. This algorithm can make full use of the computing resources of clusters but also causes high energy consumption.

### 7.3. Simulation Results

In this section, we first observe the convergence of the DDPG algorithm in the training process and then compare it with the performance of other algorithms from the perspective of a single event and the whole WSN lifetime. In the simulation, we conducted 10 groups of comparative experiments for each parameter test point. In the same group of comparison experiments, the performance of all algorithms are compared under the same node distribution and the same event set, while in different groups of comparison experiments, the node distribution and event set are different. The final result is the average of 10 experiments (The Statistical Program for Social Sciences (SPSS) was utilized to analyze the statistical significance of the experimental data by the paired sample T test. The results show that the experimental data obtained by each algorithm at each parameter test point have significant differences (*p* < 0.05).).

**Figure 5:**
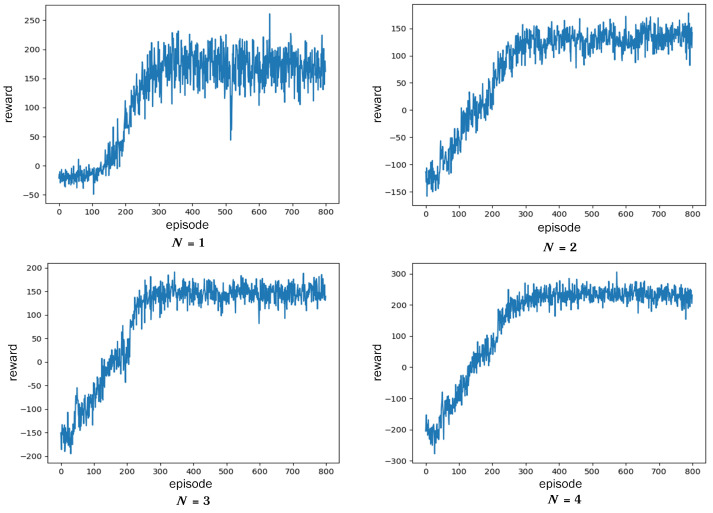
Convergence of the DDPG algorithm.

(1) The convergence of the DDPG algorithm. Figure 5 shows the reward curve of the DDPG algorithm when *N* is equal to 1, 2, 3, 4. At the beginning of training, due to the random selection of actions, a large number of tasks cannot be completed, so the reward is negative. As the number of iterations increases, the reward rises rapidly and reaches a relatively stable level after the 300th round. The fluctuation of reward value is caused by the randomness of the task bit amount $L_{ct_{z}}$ and deadline $T_{deadline}^{ct_{z}}$. Relatively, the smaller the *N*, the bigger the reward fluctuations. This is because low computing capacity makes it difficult to meet the needs of some urgent tasks.

In addition, the setting of reward parameters has an influence on whether the algorithm converges to the optimal solution. The values of parameters (μ,ν) in (16) need to be adjusted manually to help fast and stable convergence of the algorithm. A useful tip of adjustment is to firstly set the ratio of $r_{t}$ and $r_{e}$ to about 1:1 by adjusting (μ,ν), and then fine-tune it according to the convergence trend. The reference value of (μ,ν) is shown in Table 3. This table also shows the comparison of the task completion rate in one episode after the algorithm converges. As the *N* value goes down, the task completion rate will decrease due to the lack of computing capacity. When *N* = 1, the task completion rate is only 80% (lower than 95%), indicating that only one cooperative node cannot complete the event monitoring. In the process of algorithm inference, emergency tasks will be assigned to the clusters with high computing capacity (large *N* value), while the clusters with low computing capacity mainly complete lightweight tasks. Thus, the completion rate of tasks in one event can exceed 95% for the whole WSN.

**Figure 6:**
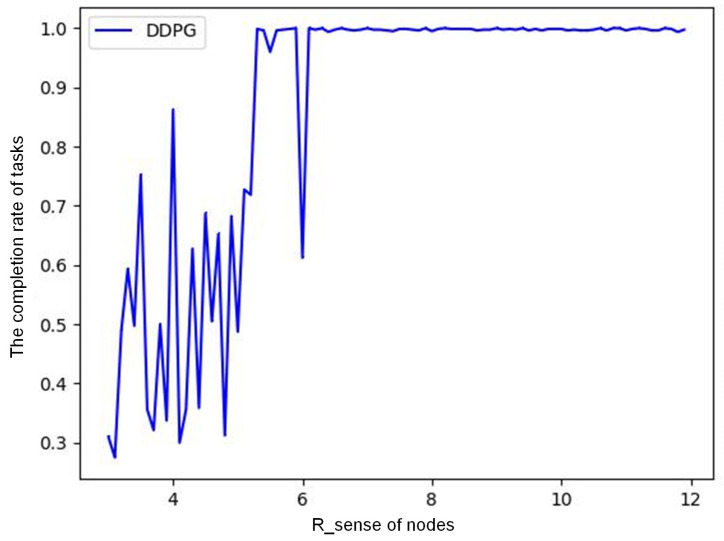
Relationship between sensing radius and task completion rate.

(2) Relationship between sensing radius and task completion rate. Figure 6 shows the relationship between the task completion rate of an event and node sensing radius $R_{sense}$ for DDPG when the monitored area is 30 × 30 m, the number of nodes is 200, and the number of tasks in a single event is 800. With the same node density, $R_{sense}$ affects the number of nodes activated by this event. If $R_{sense}$ is small, the number of activated nodes is small. If the number of tasks is huge, the nodes will die due to excessive energy consumption, and the event cannot be completed. The same is true for other competitive algorithms. More nodes should participate in cooperative computing, which can effectively improve the task completion rate. As we can see, when node density is 200/900 = 0.22 $\mathrm{node}/m^{2}$, $R_{sense}$ greater than 6.5 m can ensure that the task completion rate of a single event is more than 95%.

(3) Comparison for single event completion. In this subsection, we discuss the reaction time of the task, the task completion rate, and energy consumption of a single event in inference environment.

The reaction time of tasks: The time of event processing is divided into several uneven time slots, and one task is processed in each time slot. In addition, the main difference in each algorithm is the scheme of cooperative computing for tasks within the cluster, while for the algorithm of clustering and task allocation, they adopt the same scheme. Therefore, the reaction time of tasks is considered an important indicator of algorithm performance. Table 4 shows the average reaction time of different algorithms when the monitored area is 30 × 30 m, the number of nodes is 200, and the number of tasks is 3000. We can see that the reaction time of executing the DDPG algorithm is slightly higher than that of other algorithms but significantly lower than that of the exhaustive algorithm. The reaction time for the exhaustive algorithm to perform a computation task is about 0.5 s, which becomes unacceptable for processing events containing a large number of tasks. In addition, we can see that the reaction time of DQN is slightly less than that of the DDPG algorithm. This is because the neural network structure of the DQN algorithm is the same as that of the DDPG algorithm except for the Softmax function. Similar to the analysis in Section 6.4, it can be seen that the computational complexity of generating an action of the cluster head for DQN is $O(\sum_{c=1}^{C}n_{c}\cdot n_{c-1})$, which is slightly lower than the DDPG algorithm.

**Figure 7:**
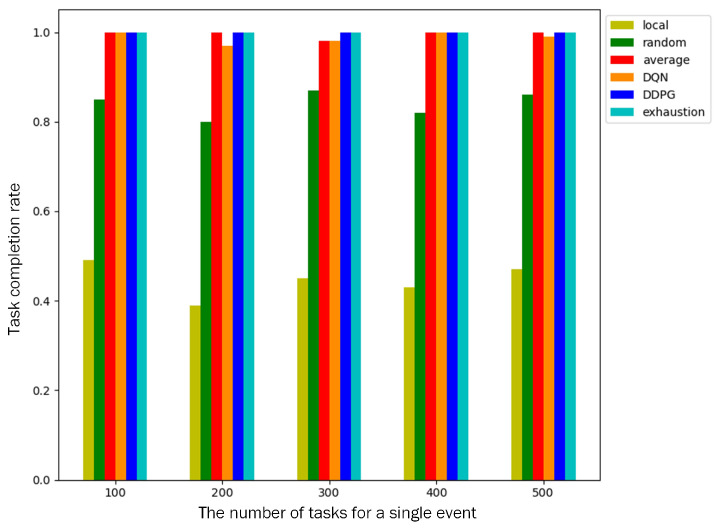
Task completion rates for a different number of tasks in an event.

Task completion rate: A total of 200 nodes are deployed in the monitored area, and a single event contains 100–500 computation tasks. We compare the task completion rates of different algorithms. As shown in Figure 7, the task completion rate of local computing is only about 40% because only cluster heads participate in the computation and cannot complete the task with a low deadline. The task completion rate of random offloading is about 80%. Both algorithms failed to complete the event because the task completion rate is less than 95%. The other four algorithms can make full use of cluster members to participate in cooperative computing, and the task completion rate of an event is above 96%.

**Figure 8:**
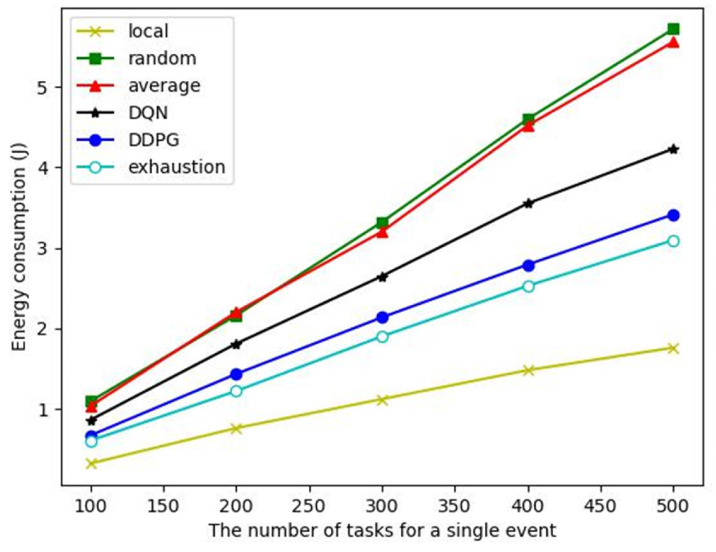
Energy consumption for a different number of tasks in an event.

Energy consumption: As shown in Figure 8, although the local computing algorithm has the lowest energy consumption, its task completion rate is only 40% (see Figure 7), which cannot complete the event monitoring. The energy consumption of the DDPG algorithm to complete a single event is close to that of the exhaustive algorithm but significantly lower than that of other comparison algorithms. The average offloading algorithm cannot dynamically offload computation according to the task and node state. Although it can ensure the completion of tasks, it consumes a lot of energy. Therefore, even though it can ensure that the event monitoring is completed, the energy consumption is very high. The action selection of the DQN algorithm is a discrete sampling, which cannot effectively cover the optimal action selection. The action space of the DDPG algorithm is continuous, so better actions can be selected to achieve a performance close to the exhaustive algorithm.

In summary, when 200 nodes are deployed, we give a comprehensive comparison of the algorithms on the reaction time of tasks, task completion rate, and energy consumption, as shown in Table 5. The proposed DDPG algorithm is superior to other algorithms and is close to the exhaustive algorithm in terms of energy consumption. At the same time, it can also ensure that the task completion rate exceeds 95% and the reaction time can be acceptable in practical applications.

(4) Comparison for the whole WSN lifetime: In the whole lifetime of the WSN, we mainly investigate the number of completed events and the number of alive nodes, which can reflect the effectiveness and energy balance of the WSN.

**Figure 9:**
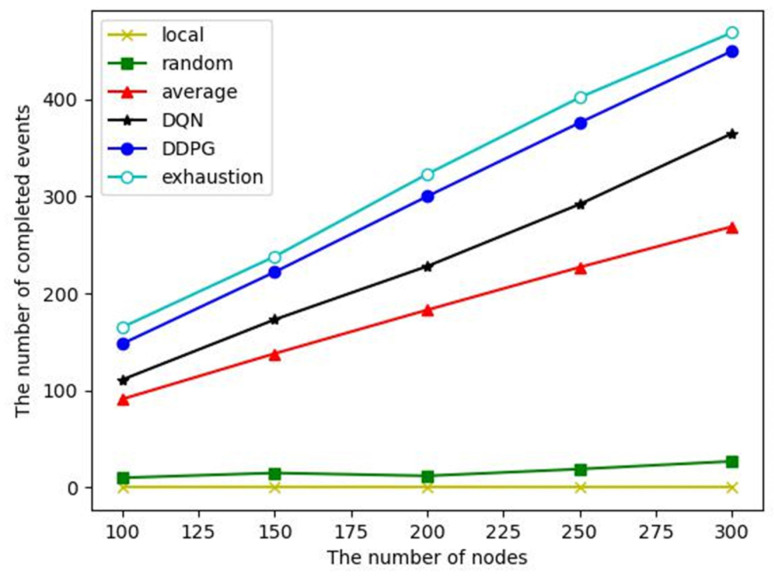
The number of completed events for a different number of nodes.

The number of completed events: Event monitoring completion is defined as a task completion rate of more than 95%. From the above analysis, it can be seen that the task completion rates of local computing and random offloading are low, so the number of completed events of them are small, as shown in Figure 9. Despite the average offloading, DQN and DDPG algorithms have similar task completion rates of a single event, and the energy consumption of the DDPG algorithm to complete an event is significantly lower than that of the other two algorithms, so it can complete more events in the whole WSN lifetime.

**Figure 10:**
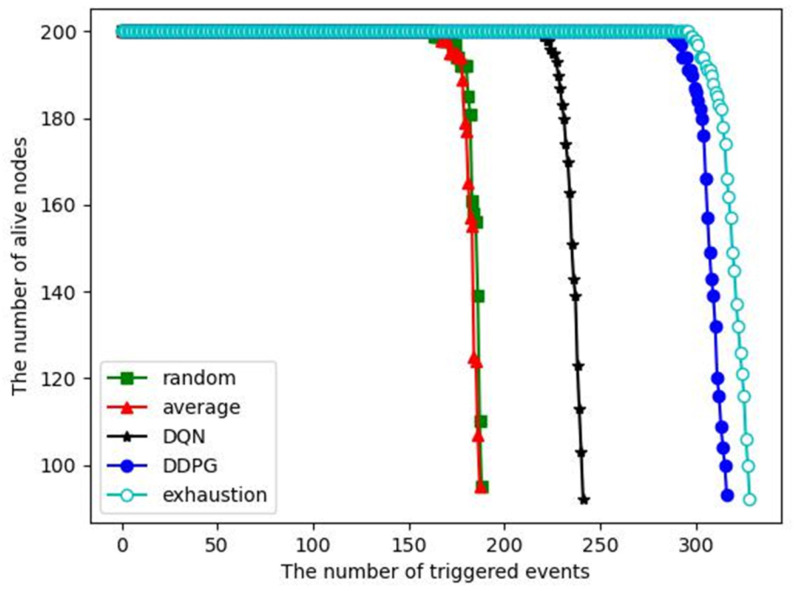
The number of alive nodes versus the number of triggered events.

The number of alive nodes versus the number of triggered events: Figure 10 shows that when 200 nodes are deployed in the monitored area, the number of alive nodes changes as the number of triggered events increases. As the number of events processed increases, the energy of the nodes decreases and some nodes may die (run out of energy). When the number of dead nodes exceeds half of the total number of nodes, we consider the entire network to have failed. Therefore, we want to have as many events processed as possible before the network crashes. In addition, if the energy consumption of each node is not balanced, some nodes will die prematurely due to taking on too many tasks, which will cause coverage holes. As we can see, the first node death only occurs at the end of the WSN lifetime for each algorithm, indicating that the dynamic clustering algorithm and inter-cluster task allocation can achieve an energy consumption balance. For the DDPG algorithm, more than 300 events are processed before the network crash, which is close to the exhaustive algorithm and significantly better than the other competitive algorithms. It indicates that the DDPG algorithm has better energy saving and energy consumption balance compared with the competitive algorithms. Local computing is not included in this comparison because it cannot complete event monitoring.

## 8. Conclusions

In this paper, in order to process emergency events locally by using the computing capacity of redundant nodes in large-scale WSNs, we firstly propose an equal-size *K*-means clustering algorithm, and then propose a DDPG-based one-to-multiple cooperative computing algorithm to obtain a computation offloading policy, which can complete more computation tasks with less energy consumption under the delay constraint. Simulation results show that the energy consumption of the proposed DDPG algorithm is more than 25% lower than that of the competitive algorithms and is close to that of the exhaustive algorithm. At the same time, it can also ensure that the task completion rate exceeds 95% and that the reaction time can be acceptable in practical applications.

## Figures and Tables

**Table 1:** Environment parameter settings.

Parameters	Value
Node initial energy	$E_{ini}=1$ J
Energy level of death	$E_{dead}=0.1$ J
Task bit amount	*L*∈ [8192, 65,536] bit
Task deadline	$T_{deadline}\in$ [300, 1000] μs
Node CPU frequency	*f* = 500 MHz
CPU cycles for 1-bit	ω = 10
Circuit energy consumption factor	$E_{elec}$ = $3\times 10^{-8}$ J/bit
Amplifier energy consumption factor	$\xi_{amp}=4\times 10^{-10}$ J/bit/$m^{2}$
Computation energy consumption factor	$\kappa =10^{-24}$
Transmission rate	*C* = 50 M bit/s
Node sensing radius	$R_{sense}$ = 9 m

**Table 2:** Neural network hyperparameter settings.

Parameters	Value
Number of Iterations	episode=800
Learning rate of actor network	$\alpha_{a}$ = 0.001
Learning rate of critic network	$\alpha_{c}$ = 0.002
The size of experience pool	*buffer* = 500,000
The size of the sample	*batch* = 128
Reward discount factor	γ = 0.9
The soft update speed	τ = 0.005

**Table 3:** Parameters and task completion rate for different *N*.

The Value of *N*	μ	ν	Task Completion Rate
4	1.4	500	99%
3	1.3	450	97%
2	1.2	350	95%
1	0.8	350	80%

**Table 4:** Comparison of the reaction time of tasks.

Algorithm	Reaction Time
Local	$8.42\times 10^{-5}$ s
Random	$8.61\times 10^{-5}$ s
Average	$9.45\times 10^{-5}$ s
DQN	$32.7\times 10^{-5}$ s
DDPG	$36.3\times 10^{-5}$ s
Exhaustion	0.538 s

**Table 5:** Comparison of the reaction time of tasks, task completion rate, and energy consumption.

Algorithm	Reaction Time	Task Completion Rate	Energy Consumption
Local	$8.42\times 10^{-5}$ s	39%	0.71 J
Random	$8.61\times 10^{-5}$ s	79%	2.16 J
Average	$9.45\times 10^{-5}$ s	99%	2.20 J
DQN	$32.7\times 10^{-5}$ s	98%	1.85 J
DDPG	$36.3\times 10^{-5}$ s	99%	1.37 J
Exhaustion	0.538 s	99%	1.22 J

## Data Availability

Not applicable.
